# Mechanisms of Toxicity of Ag Nanoparticles in Comparison to Bulk and Ionic Ag on Mussel Hemocytes and Gill Cells

**DOI:** 10.1371/journal.pone.0129039

**Published:** 2015-06-10

**Authors:** Alberto Katsumiti, Douglas Gilliland, Inmaculada Arostegui, Miren P. Cajaraville

**Affiliations:** 1 CBET Research Group, Department of Zoology and Animal Cell Biology, Faculty of Science and Technology and Research Centre for Experimental Marine Biology and Biotechnology PIE, University of the Basque Country UPV/EHU, Plentzia, Spain; 2 European Commission–Joint Research Centre, Institute of Health and Consumer Protection, NSB Unit, Ispra (VA), Italy; 3 Department of Applied Mathematics, Statistics and Operations Research, Faculty of Science and Technology, University of the Basque Country UPV/EHU, Leioa, Spain; University of California, Merced, UNITED STATES

## Abstract

Silver nanoparticles (Ag NPs) are increasingly used in many products and are expected to end up in the aquatic environment. Mussels have been proposed as marine model species to evaluate NP toxicity *in vitro*. The objective of this work was to assess the mechanisms of toxicity of Ag NPs on mussel hemocytes and gill cells, in comparison to ionic and bulk Ag. Firstly, cytotoxicity of commercial and maltose stabilized Ag NPs was screened in parallel with the ionic and bulk forms at a wide range of concentrations in isolated mussel cells using cell viability assays. Toxicity of maltose alone was also tested. LC50 values were calculated and the most toxic Ag NPs tested were selected for a second step where sublethal concentrations of each Ag form were tested using a wide array of mechanistic tests in both cell types. Maltose-stabilized Ag NPs showed size-dependent cytotoxicity, smaller (20 nm) NPs being more toxic than larger (40 and 100 nm) NPs. Maltose alone provoked minor effects on cell viability. Ionic Ag was the most cytotoxic Ag form tested whereas bulk Ag showed similar cytotoxicity to the commercial Ag NPs. Main mechanisms of action of Ag NPs involved oxidative stress and genotoxicity in the two cell types, activation of lysosomal AcP activity, disruption of actin cytoskeleton and stimulation of phagocytosis in hemocytes and increase of MXR transport activity and inhibition of Na-K-ATPase in gill cells. Similar effects were observed after exposure to ionic and bulk Ag in the two cell types, although generally effects were more marked for the ionic form. In conclusion, results suggest that most observed responses were due at least in part to dissolved Ag.

## Introduction

Silver nanoparticles (Ag NPs) are emerging as one of the fastest growing product categories in the nanotechnology industry. Due to their physico-chemical properties, including a high thermo-electrical conductivity, catalytic activity and non-linear optical behavior [1, 2] Ag NPs have potential value in the formulation of inks, microelectronic products and medical imaging devices [3]. However, it is their exceptional broad-spectrum bacteriocidal property [3, 4] that makes Ag NPs extremely popular in a diverse range of consumer goods.

Worldwide, the production of Ag NPs is estimated at about 55 t/y [5] and volume of production is expected to increase significantly in the next years. Ag NPs may be released to the environment by different routes, including during their synthesis, incorporation into goods and from consumer products, reaching wastewaters and finally the aquatic environment [4]. The estimated concentration of Ag NPs in aquatic environments is about 0.01 μg/L [6] and exponential increases are predicted due to the increased usage and consequent discharge levels.

Among the vast number of NPs available, Ag NPs are of special concern for the aquatic environment since silver is known to be one of the most toxic metals to organisms, showing effects at very low concentrations (ng.L^-1^ range) [4]. Toxicity of Ag NPs has been reported in a variety of aquatic organisms including algae [7, 8], daphnids [8], ragworm [9], bivalve mollusks [9–13] and fishes [8, 14, 15].

In bivalves, bioaccumulation of Ag and adverse effects such as alterations in genes related with metal detoxification/metabolism regulation, in antioxidant capacity and in embryo development have been reported after exposure to Ag NPs [9–13, 16–18]. Al-Sid-Cheikh et al. [18] found that scallops (*Chlamys islandica*) can accumulate significant quantities of Ag NPs in a short time followed by an efficient depuration process. In the mussel *Mytilus edulis*, exposed to radio-labelled Ag NPs (<40 nm, 0.7 mg/L), 60% accumulated in the soft tissues with maximum concentration in the digestive gland, whilst some 7% was found in the mussel’s extrapallial fluid [16]. Ringwood et al. [10] reported significant increases in metallothionein mRNA levels of embryos and adult oysters (*Crassostrea virginica*) exposed to Ag NPs. Based on proteomic analysis, Gomes et al. [12] showed that Ag NPs toxicity in the mussel *Mytilus galloprovincialis* is mediated by oxidative stress-induced cell signaling cascades that can lead to cell death. Similarly, Buffet et al. [13] reported increases in antioxidant enzyme activities (catalase, glutathion S-transferase, superoxide dismutase) in the endobenthic bivalve *Scrobicularia plana* exposed for 14 days to Ag NPs via water or diet (microalgae). Ag NPs have been also reported to cause genotoxicity. In *M*. *galloprovincialis* Ag NPs induced DNA damage in hemolymph cells [11]. Adverse effects on embryonic development and lysosomal destabilization were reported in adult *C*. *virginica* exposed to Ag NPs [10].

The *in vivo* studies summarized above have reported several mechanisms of toxicity of Ag NPs in bivalves. *In vitro* techniques could provide valuable tools to rapidly screen the toxicity of different types of Ag NPs and to identify additional cellular mechanisms altered by the exposure to Ag NPs. Mussel hemocytes are hemolymph cells responsible for the immune defense of mollusks [19, 20] and constitute important targets for NP toxicity [21–27]. Mussel gill cells have also been proved to be a suitable *in vitro* epithelial cell model for screening the potential cytotoxicity of NPs [25–28] and for the study of cellular mechanisms of toxicity of NPs [27] due to their role in nutrient uptake and digestion and in respiration [29].

A concentration-dependent lysozyme release and extracellular oxyradical and nitric oxide production were found in mussel hemocytes exposed *in vitro* to carbon black nanoparticles [21] and to C60 fullerenes, TiO_2_ and SiO_2_ NPs [22]. Ciacci et al. [23] demonstrated that different metal oxide NPs (TiO_2_, SiO_2_, ZnO, CeO_2_) rapidly elicited immune responses in mussel hemocytes *in vitro*, depending not only on NP concentration, but also on particle chemistry and behaviour in media. Cytotoxicity of metal-bearing NPs (ZnO, SiO_2_, Au) was screened in mussel hemocytes and gill cells and results showed that properties of NPs such as size, shape and ability to release of metal ions influence their toxicity [25]. Different types of TiO_2_ NPs were also cytotoxic to mussel hemocytes and gill cells, and cytotoxicity varied according to the mode of synthesis, crystalline structure and size of NPs and also was influenced by presence of additives [26]. Exposure to CdS quantum dots activated cellular mechanisms leading to oxidative stress, DNA damage and increased lysosomal and MXR transport activities in both hemocytes and gill cells of mussels [27]. To the best of our knowledge, no studies have been performed with the objective of elucidating the cytotoxicity and mechanisms of action of Ag NPs in mussel cells *in vitro*.

In this context, the aims of the present work were: 1) to screen the cytotoxicity of a set of Ag NPs of different sizes in parallel with ionic and bulk Ag using cell viability tests and 2) to compare the mechanisms of action of Ag NPs with those of the ionic and bulk forms using an array of functional tests covering the main cellular processes in hemocytes and gill cells of mussels.

## Materials and Methods

### Ag NPs, ionic and bulk forms

Maltose-stabilized Ag NPs suspensions (Ag20-Mal, Ag40-Mal and Ag100-Mal) were obtained from the JRC (EC Joint Research Center, Ispra) in form of suspensions. Commercial Ag NPs (Ag20 and Ag80) were purchased from Nanostructured & Amorphous materials, Inc. (Houston, USA) in form of powder. Ionic Ag (Ag in 2% nitric acid) was purchased from BDH PROLABO (Barcelona, Spain) and bulk Ag (10μm, ≥99.9% trace metals basis) was purchased from Sigma Aldrich (St. Louis, USA).

Synthesis of maltose-stabilized Ag NPs and characterization of Ag NPs and bulk Ag Maltose-stabilized Ag NPs suspensions were synthesized using the Tollens method [30] through the reduction of the complex cation [Ag(NH_3_)_2_]^+^ by sugars. Briefly, NH_4_OH was added to 100 mL of 2 mM aqueous AgNO_3_ solution under vigorous stirring to form the ammonia sugar complex. Subsequently, 100mL aqueous solution of 0.01 M D-(+)-maltose monohydrate was added to the mixture. The reduction reaction of the silver by the sugar was then initiated by adding NaOH solution to increase the pH to approximately 11. The experiments were performed at room temperature (approx. 25°C) in a 250mL flask protected from light. By changing the concentrations of ammonium hydroxide and the sodium hydroxide used in the reaction mixture it was possible to vary in a controlled manner the final size of particles produced. In particular the production of the 40 nm particles required 0.4 mL of 4N NH_4_OH and 5.5 mL of 1 M NaOH while 100 nm particles required 0.5 mL of 4N NH_4_OH and 2 mL of 0.1 M NaOH.

Ag NPs and bulk Ag were characterized through Transmission Electron Microscopy (TEM, Hitachi H7100) or Scanning Electron Microscopy (SEM, Philips XL30) to determine particles’ size and shape. Additional data on commercial Ag NPs are available in the manufacturer’s webpage (http://www.nanoamor.com). For maltose-stabilized Ag NPs UV-visible absorption spectroscopy was used to measure the position of the plasmon resonance absorption bands, which are highly characteristic of these metallic nanoparticles. Dynamic Light Scattering (DLS, Malvern Zetasizer Nano ZS instrument) was used to determine particle size distribution/aggregation and zeta potential. In addition, abiotic reactivity of Ag NPs was studied. Dissolution of Ag NPs was assessed in artificial seawater following Misra et al. [31]. Briefly, 0.1 mM of Ag NP suspensions were added to a dialyzer tube and immersed in seawater for up to 7 days. Samples were periodically extracted from the solution and retained for later trace metal analysis using ICP-MS (Agilent 7700 ICP-MS instrument) in different sampling times: from minimum of 1 h up to 7 days (168 h) maximum exposure.

### Isolation of mussel cells

Mussels *Mytilus galloprovincialis* Lmk. of 3.5–4.5 cm shell length were collected from Mundaka, Gulf of Biscay (43°24'16"N; 2°41'43"W), a relatively non-polluted area [32–34]. Permission to sample mussels in the Basque coast is obtained annually from the Fisheries and Aquaculture Direction of the Basque Government (last permission issued 10th June 2014, registry number 221670). Mussels were acclimatized for 2 days at 16–18°C, constant aeration and daily food supply in the aquaria facilities of the Cell Biology in Environmental Toxicology (CBET) research group at UPV/EHU before cell isolation.

Mussels’ hemocytes were isolated according to Gómez-Mendikute and Cajaraville [35] with modifications. Briefly, hemolymph of 50 animals was withdrawn from the posterior adductor muscle, pooled and diluted at 2 x 10^5^ cells/mL (> 95% viable according to trypan blue exclusion assay) in anti aggregation solution (171 mM NaCl; 0.2 M Tris; 0.15% v/v HCl 1 N; 24 mM EDTA) under aseptic conditions in a vertical laminar airflow cabinet (Cultair BC100, Cultek S.L., Madrid, Spain). Cell suspensions (200 μL) were seeded into six replicates of 96-well microplates in culture medium (Basal Medium Eagle, 1040 mOsm/kg, pH 7.4, supplemented with 0.001% gentamicin). Microplates were centrifuged (Beckman Coulter, Palo Alto, USA) at 270 x g for 10 min at 4°C in order to favour cells to attach.

Gill cells were isolated according to Venier et al. [36] with modifications. Briefly, gills were excised under the aseptic conditions described above and washed twice for 1 h in saline solution supplemented with 10 U/mL bacitracin, 400 U/mL polymyxin B, 20 μg/mL ampicillin, 300 U/mL penicillin G, 300 U/mL streptomycin, 50 μg/mL amphotericin B and 50 U/mL nystatin. Afterwards, gills were enzymatically digested with 0.6–2.4 U/mL dispase II (Roche Diagnostics GmbH, Mannheim, Germany) for 10 min at room temperature, filtered (280 μm and 100 μm nets), washed twice by centrifugation at 270 x *g* for 10 min at 4°C and resuspended in Alsever´s solution. Cells were then diluted (5 x 10^5^ cells/mL, > 95% viable according to trypan blue exclusion assay) and seeded into six replicates of 96-well microplates in culture medium (Leibovitz L-15 medium, 1040 mOsm/kg, pH 7.4, supplemented with 1 mg/mL glucose, 50 μg/mL glucosamine, 1.7 mg/mL Hepes, 100 U/mL penicillin, 100 μg/mL streptomycin, 100 μg/mL neomycin and 100 μg/mL kanamycin).

Before performing the exposures, both hemocytes and gill cells were maintained for 24 h in supplemented media at 18°C in a Sanyo incubator (Osaka, Japan) to establish the primary cell cultures.

### *In vitro* exposures

A two-tier procedure was employed for the *in vitro* toxicity assessment. In the first tier, mussel cells were exposed to a wide range of concentrations (0.001, 0.01, 0.1, 1, 10, 25, 50 and 100 mg Ag/L) of maltose stabilized and commercial Ag NPs, bulk Ag and ionic Ag in order to assess cytotoxicity through cell viability assays. Cytotoxicity of maltose was also tested. LC50 values were calculated and the most toxic Ag NPs were selected for in-depth mechanistic studies in the second tier. For this, mussel cells were exposed to sublethal concentrations (below LC25 for each Ag form) of Ag NPs (0.15, 0.31, 0.62, 1.25 and 2.5 mg Ag/L), bulk Ag (0.62, 1.25, 2.5, 5 and 10 mg Ag/L) and ionic Ag (0.03, 0.06, 0.12, 0.25 and 0.5 mg Ag/L) in order to evaluate the mechanisms involved in their toxicity through a series of functional tests. All exposures were performed for up to 24 h.

### Cell viability assays

For the neutral red (NR) assay, retention of the cationic dye neutral red in viable cells was assessed as reported previously [37]. The assay is based on the incorporation of the dye into the lysosomes of living cells [38]. After 1 h incubation with NR solution (0.04%, pH 7.3–7.4) in order to allow the uptake of the dye, cells were centrifuged at 270 x *g* for 10 min at 4°C and washed several times with PBS to eliminate non incorporated dye. Afterwards, dye was extracted from intact cells with an acetic acid (0.5%) ethanol (50%) solution. In order to avoid the interference of NPs with spectrophotometric measurements, samples were transferred to V bottom 96-well microplates and centrifuged at 270 x *g* for 30 min at 4°C. Supernatants were then placed in flat bottom 96-well microplates and absorbance was determined at 550 nm in a Biotek EL 312 microplate spectrophotometer reader (Winooski, USA).

The thiazolyl blue tetrazolium bromide (MTT) assay was used following manufacturer’s instructions (Sigma Aldrich, M5655) with modifications. The assay is based on the capacity of actively respiring cells to convert water-soluble MTT into a non-soluble purple formazan. After 2.5 h incubation of the cells with MTT solution (5 mg/mL), resulting product (formazan) was extracted from living cells with dimethyl sulphoxide (DMSO) for 1 h, transferred to V bottom 96-well microplates and centrifuged at 270 x *g* for 30 min at 4°C. Supernatants were then placed in flat bottom 96-well microplates and optical density was read at 495 nm in the same microplate spectrophotometer described above.

### Reactive oxygen species (ROS) production

ROS production was detected in mussel cells using the kit Reactive Oxygen Species (ROS) Detection Reagents (Invitrogen, C400) following manufacturer’s instructions with modifications. Mussel cells were incubated with 2′,7′-dichlorofluorescein (DCF) and calcein for 30 min, washed twice with PBS and maintained in cell culture media up to analysis. In order to avoid the interference of NPs with fluorometric measurements, samples were transferred to V bottom 96-well microplates and centrifuged at 270 x *g* for 30 min at 4°C. Supernatants were then placed in flat bottom 96-well microplates and fluorescence was detected at excitation 492–495/emission 517–527 nm using a Bio-Tek FLx 800 microplate fluorimeter reader (Winooski, USA). ROS production was monitored after 1, 3, 6 and 24 h exposure.

### Catalase (CAT) activity

CAT activity was quantified following Aebi [39] with modifications, as the decrease in absorbance at 240 nm due to the H_2_O_2_ consumption (20 mM H_2_O_2_ in 50 mM phosphate buffer pH 7). Absorbances were read every 20 s for 5 min in UV/VIS 96-well microplates in the same spectrophotometer described previously.

### Comet assay

Comet assay was performed following Raisuddin and Jha [40] with some modifications. Cells treated with 50 μM H_2_O_2_ were used as positive control. After *in vitro* exposures, cells were trypsinized and resuspended in 0.5% low melting point (LMP) agarose. Two drops (85 μL each) of the cell suspension were placed on slides coated with 1% normal melting point agarose. Slides were kept on ice for 10 min in order to allow LMP solidification. Slides were immersed in chilled lysing solution (2.5 M NaCl, 100 mM EDTA, 10 mM Tris base, 1% N-lauroyl-sarcosine, 1% Triton X-100, and 10% DMSO; adjusted to pH 10 with 0.5% NaOH) for 1 h at 4°C in the dark to remove cellular proteins. The slides were then washed with distilled water, transferred to an electrophoresis tank containing 1 N NaOH and 200 mM EDTA (pH 13) and kept for 20 min to permit alkaline DNA unwinding. Electrophoresis was carried out for 30 min (300 mA, 25 V) and afterwards, slides were removed from the electrophoresis chamber, treated with neutralization buffer (0.4 M Tris-HCl buffer, pH 7.5) and fixed for 3 min with chilled methanol. For analysis, slides were stained with ethidium bromide (2 μg/mL in distilled water), and observed under an Olympus BX61 fluorescence microscope (Olympus optical Co, Hamburg, Germany). 50 randomly selected cells were analyzed from each slide (25 in each gel from duplicate cultures) and scored using the Komet 5.5 image analysis system (Kinetic Imaging, Liverpool, UK).

### Acid phosphatase (AcP) activity

The lysosomal AcP activity was quantified following Olabarrieta et al. [41] Briefly, cells were incubated with 2 mg/mL of p-nitrophenylphosphate disodic salt in citrate buffer (pH 5) for 1 h at 16°C. Afterwards, the reaction was stopped by adding 0.25 N NaOH. Samples were transferred to V bottom 96-well microplates and centrifuged at 270 x *g* for 30 min at 4°C. Supernatants were then placed in flat bottom 96-well microplates and absorbance of the yellow colour product formed in the alkaline medium was quantified at 405 nm in the same spectrophotometer described above.

### Multixenobiotic resistance (MXR) transport activity

The MXR transport activity was quantified following manufacturers’ instructions. This assay is based on the principle that cells expressing high levels of P-glycoproteins (Pgp) rapidly extrude non-fluorescent calcein AM from the cytosol across the plasma membrane, reducing accumulation of fluorescent calcein in the cytosol. Briefly, cells were incubated with 1 μM calcein AM (Vybrant Multidrug Resistance assay kit, Molecular Probes, Oregon, USA) for 30 min at 18°C in darkness. Fluorescence of the intracellular calcein was quantified at excitation 485/emission 516 nm in the same microplate fluorescence reader described before.

### Na-K-ATPase activity

Na-K-ATPase activity was quantified only in gill cells following Muscella et al. [42] with modifications. After exposures, gill cells were immediately permeabilized by freezing for 10 min at—20°C. The reaction was started by adding the reaction mixture (20 mM KCl, 8 mM MgCl_2_, 100 mM NaCl, 0.5 mM EGTA, 40 mM Tris, 10 mM phosho-enol pyruvate, 0.25 mM NADH, 1 mM fructose-1,6-diphosphate, 5 mM ATP, 1.1 U/mL lactate dehydrogenase, 0.9 U/ml pyruvate kinase, with or without 1 mM ouabain). Absorbance was read at 340 nm, taken at 1-min intervals in the same spectrophotometer described above. The slope of the disappearance curve of NADH represents the ATP hydrolysis rate. To obtain the Na-K-ATPase activity, the slope of the activity in the presence of ouabain (ouabain-resistant ATPase activity) was subtracted from the slope obtained in the absence of ouabain (total ATPase activity).

### Actin cytoskeleton morphology

Actin cytoskeleton integrity was evaluated only in hemocytes according to Chazotte [43]. After *in vitro* exposures, hemocytes were fixed with methanol for 10 min, permeabilized with acetone for 20 sec and incubated with tetramethylrhodamine B isothiocyanate (TRITC)-conjugated phalloidin for 30 min in the dark. Cells were then washed several times with PBS, and observed under an Olympus Fluorview FV500 confocal microscope (Hamburg, Germany) at excitation 540-545/emission 570–573 nm.

### Phagocytic activity

Phagocytic activity was measured only in hemocytes by the ability of cells to phagocytose neutral red (NR)-stained zymosan [44]. Following incubation with NR-stained zymosan particles for 30 min at 18°C, cells were fixed with methanol for 20 min. NR was extracted from the internalized zymosan particles with an acetic acid ethanol solution. Extracts were transferred to V bottom 96-well microplates and centrifuged at 270 x *g* for 30 min at 4°C. Supernatants were then placed in flat bottom 96-well microplates and absorbance was determined as described above for NR cell viability assay.

### Statistics

Bootstrap resampling techniques [45] were used to assess differences between *in vitro* activities of control (non-treated) and treated mussel cells. For each experiment, N = 2000 repetitions of the same size of the original sample were selected by bootstrap resampling. After that, Bonferroni´s correction was used for multiple comparisons. Significance level was globally stated at 5%. Six replicates were performed for all tests except for catalase activity, where four replicates were used, and for Comet assay, where fifty cells were analyzed per experimental group. Bootstrap analyses were performed using the SAS 9.2 software (Cary, USA). LC50 values were calculated through Probit analysis using the SPSS 17.0 software (Chicago, USA).

## Results

### Ag NPs and bulk Ag characterization

**Fig 1:**
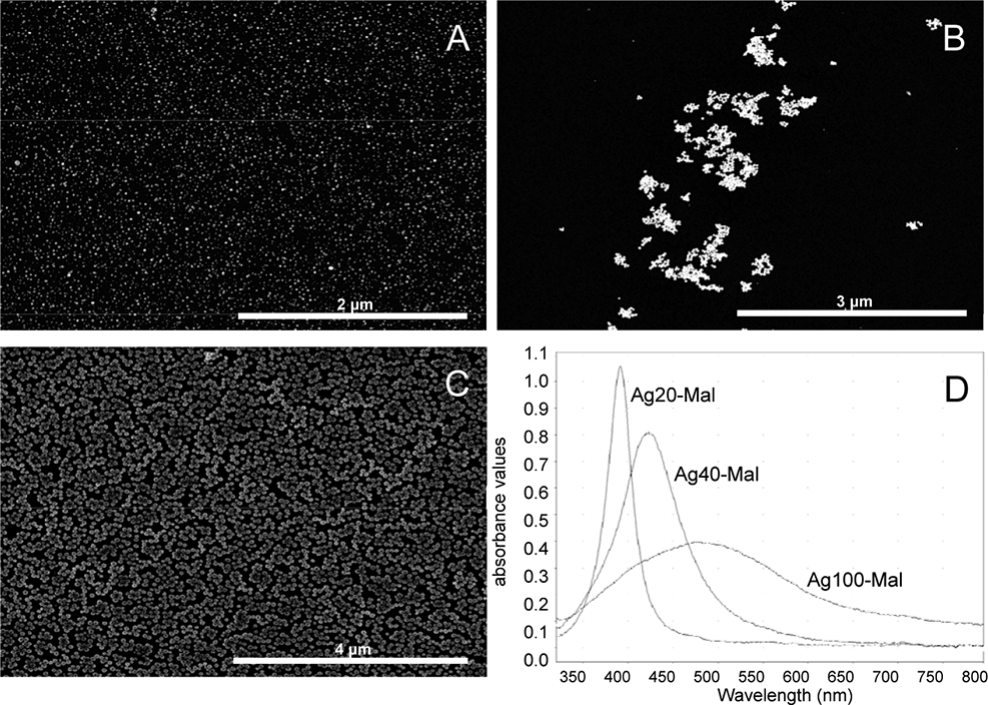
SEM images and UV absorption spectrum of the maltose-stabilized Ag NPs. SEM images of Ag20-Mal (A), Ag40-Mal (B) and Ag100-Mal (C) NPs and their respective UV absorption spectrum (D) in distilled water.

**Fig 2:**
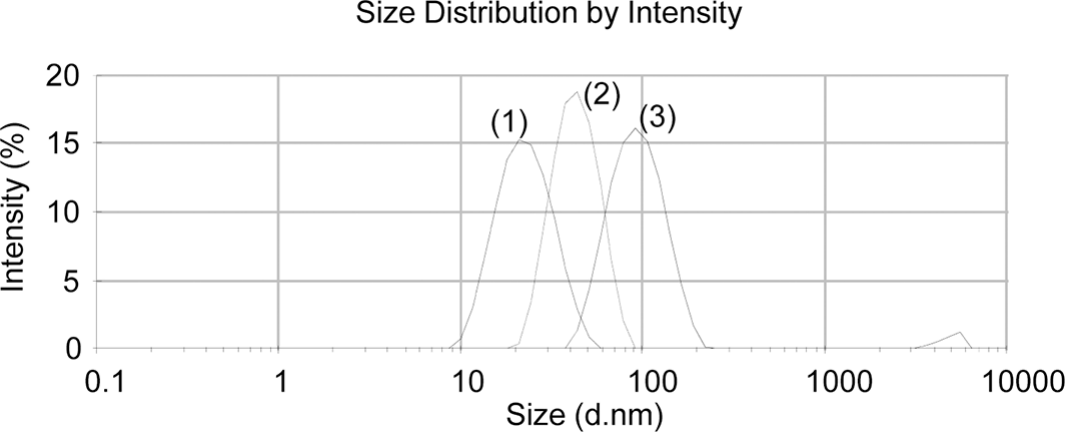
DLS patterns of size distribution of the maltose-stabilized Ag NPs. Ag20-Mal (1), Ag40-Mal (2) and Ag100-Mal (3) NPs in distilled water. d.nm = diameter in nm.

**Fig 3:**
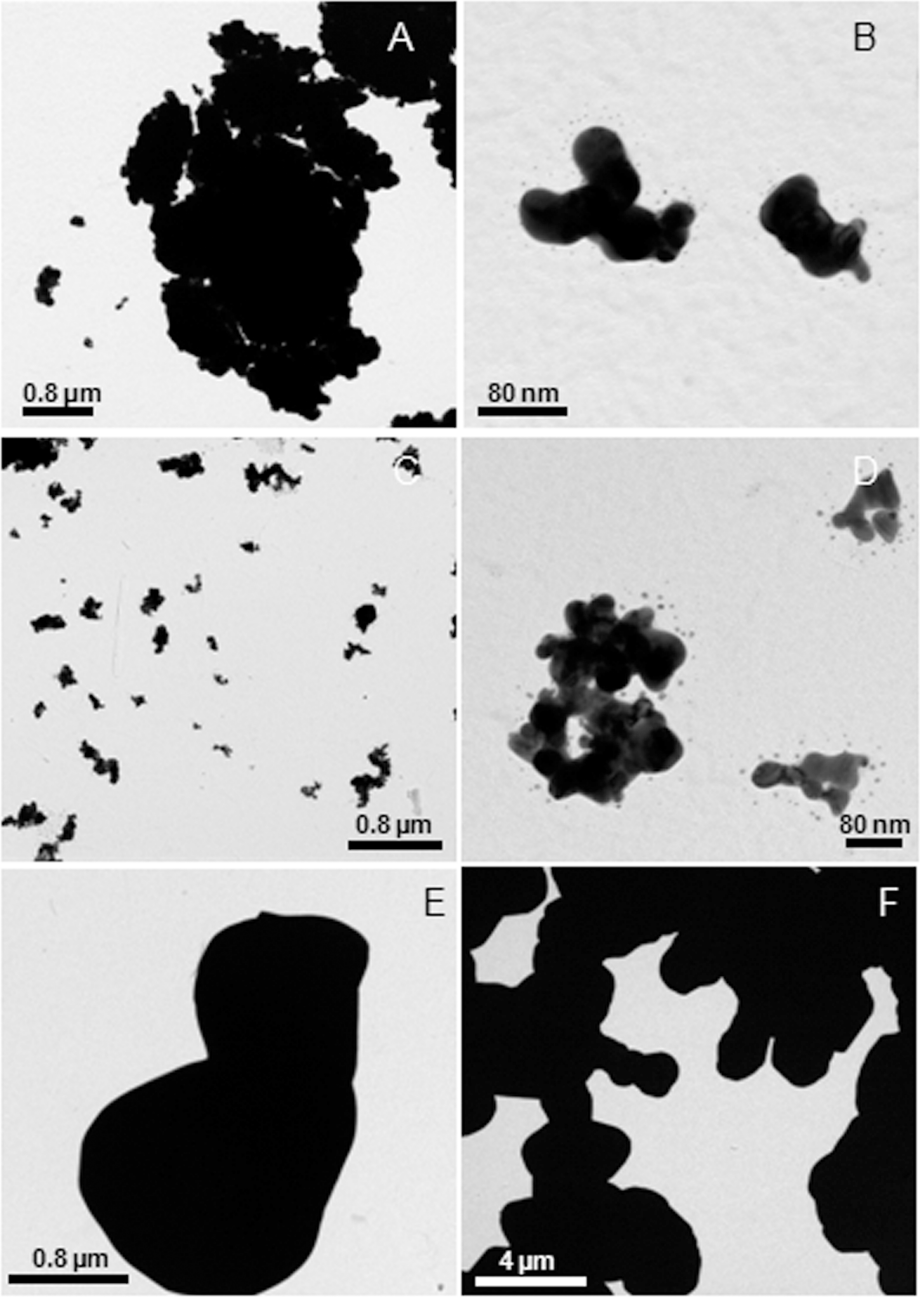
TEM images of the commercial Ag NPs. Ag20 NPs (A and B), Ag80 NPs (C and D) and bulk Ag (E and F).

According to the SEM analysis, Ag20-Mal, Ag40-Mal and Ag100-Mal samples correspond to spherical NPs of roughly 20, 40 and 100 nm (Fig 1A–1C). The absorption wavelength peaks of maltose-stabilized Ag NPs (Fig 1D) were consistent with NPs of the size diameters observed in the SEM analysis. DLS analysis showed that maltose-stabilized Ag NPs of the three sizes were monodispersed NPs (Fig 2), with negative zeta potential values ranging from -30 to -35 mV, which correspond to colloidally stable samples. TEM analysis of the commercial Ag20 and Ag80 NPs showed that both samples contain spherical (below 10 nm) and larger polydispersed NPs of roughly 20 and 80 nm respectively besides of some aggregated NPs (Fig 3A–3D). TEM images of bulk Ag showed that sample contains larger amorphous Ag particles over 2 μm in size (Fig 3E and 3F). Ag20 NPs, Ag80 NPs and bulk Ag showed high zeta potential values (-50±0.5, -44±1, -68±3 mV respectively) that correspond to stable electrostatically stabilized particles in dispersions. Dissolution of maltose stabilized Ag NPs in SW was detected after less than 1 h of experimentation (Table 1). The release of silver ions was rapid and continuous. During the first 24 h, Ag20-Mal NPs tended to dissolve faster than the other two maltose stabilized Ag NPs (Table 1). At 24 h, 11.7% of the total Ag20-Mal NPs was converted into ionic Ag when only 8.7 and 7.2% of Ag40-Mal and Ag100-Mal respectively were ionized (Table 1). More than 20% of the metallic silver was converted into ionic form at 168 h in SW (Table 1).

**Table 1:** Temporal release of Ag ions as % of total starting mass of nanoparticle material in artificial seawater containing a 0.1 mM maltose-stabilized Ag NPs suspension.

Time (h)	Ag20-Mal	Ag40-Mal	Ag100-Mal
**0.5**	2.6	1.4	1.0
**1**	3.4	1.6	1.0
**2**	4.1	2.2	1.6
**4**	5.3	2.9	2.3
**24**	11.7	8.7	7.2
**48**	16.6	14.1	11.7
**72**	20.6	19.7	15.1
**168**	27.8	34.6	21.3

### Cell viability

**Fig 4:**
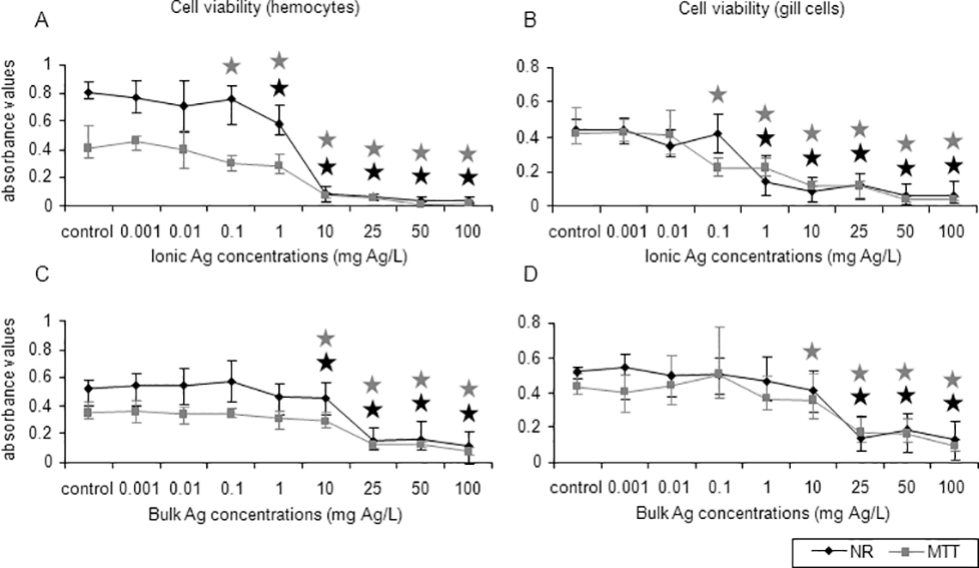
Effects of ionic and bulk Ag on mussel cells viability. Cell viability (NR and MTT assays) in mussel hemocytes and gill cells exposed to ionic Ag (A and B) and bulk Ag (C and D) for 24 h. Data are given as absorbance values (means ± confidence intervals). Stars indicate significant differences (p<0.05) in treated cells with respect to controls according to the bootstrap analysis followed by Bonferroni’s correction. Black stars correspond to the NR assay and grey stars to the MTT assay. *n* = 6 replicates per treatment.

Based on the NR and MTT assays, all Ag forms were cytotoxic to mussel hemocytes and gill cells. Ionic Ag was the most toxic Ag form tested (Fig 4A and 4B). In the two cell types, cytotoxicity started at 0.1 mg Ag/L (26–46% decrease, MTT assay) and at 1 mg Ag/L (27–67% decrease, NR assay) (*p*<0.05) (Fig 4A and 4B). LC50 values for hemocytes and gill cells exposed to ionic Ag were lower than 1.2 mg Ag/L, indicating a strong cytotoxicity (Table 1). Bulk Ag decreased cell viability in hemocytes (13–18% decrease, depending on the test) starting at a concentration of 10 mg Ag/L (Fig 4C) and in gill cells at 25 mg Ag/L (73% decrease, NR assay) and 10 mg Ag/L (19% decrease, MTT assay) (*p*<0.05) (Fig 4D). LC50 values for bulk Ag were in the range 17.8–21 mg Ag/L for both cell types (Table 1).

**Fig 5:**
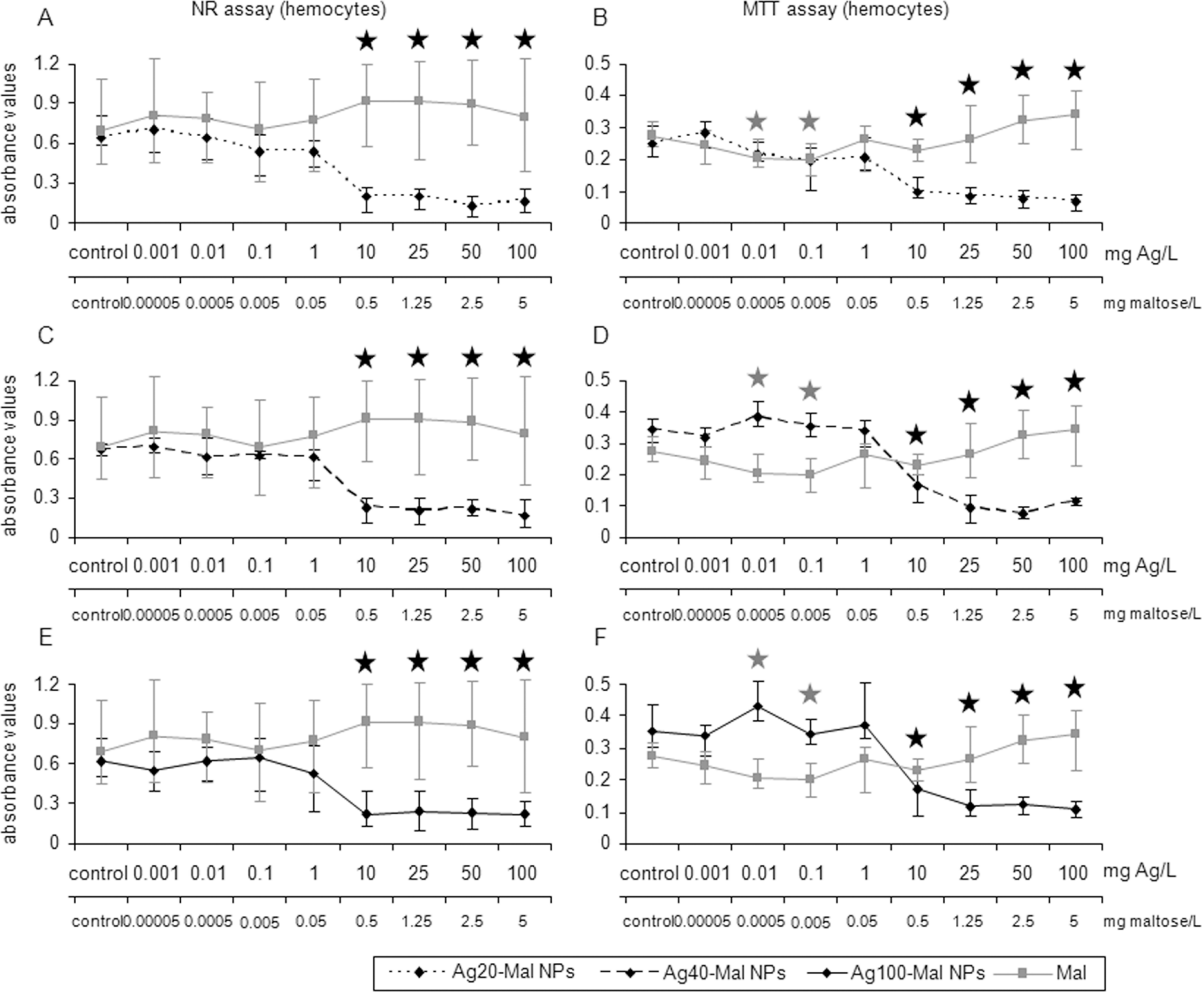
Effects of maltose-stabilized Ag NPs on hemocytes viability. Cell viability based on NR and MTT assays in mussel hemocytes exposed to Ag20-Mal (A and B), Ag40-Mal (C and D) and Ag100-Mal (E and F) NPs and to pure maltose (at the same concentrations present in corresponding maltose-stabilized Ag NPs suspensions) for 24 h. Data are given as absorbance values (means ± confidence intervals). Stars indicate significant differences (p<0.05) in treated cells with respect to controls according to the bootstrap analysis followed by Bonferroni’s correction. Black stars correspond to the maltose-stabilized Ag NPs and grey stars to the maltose results. *n* = 6 replicates per treatment.

**Fig 6:**
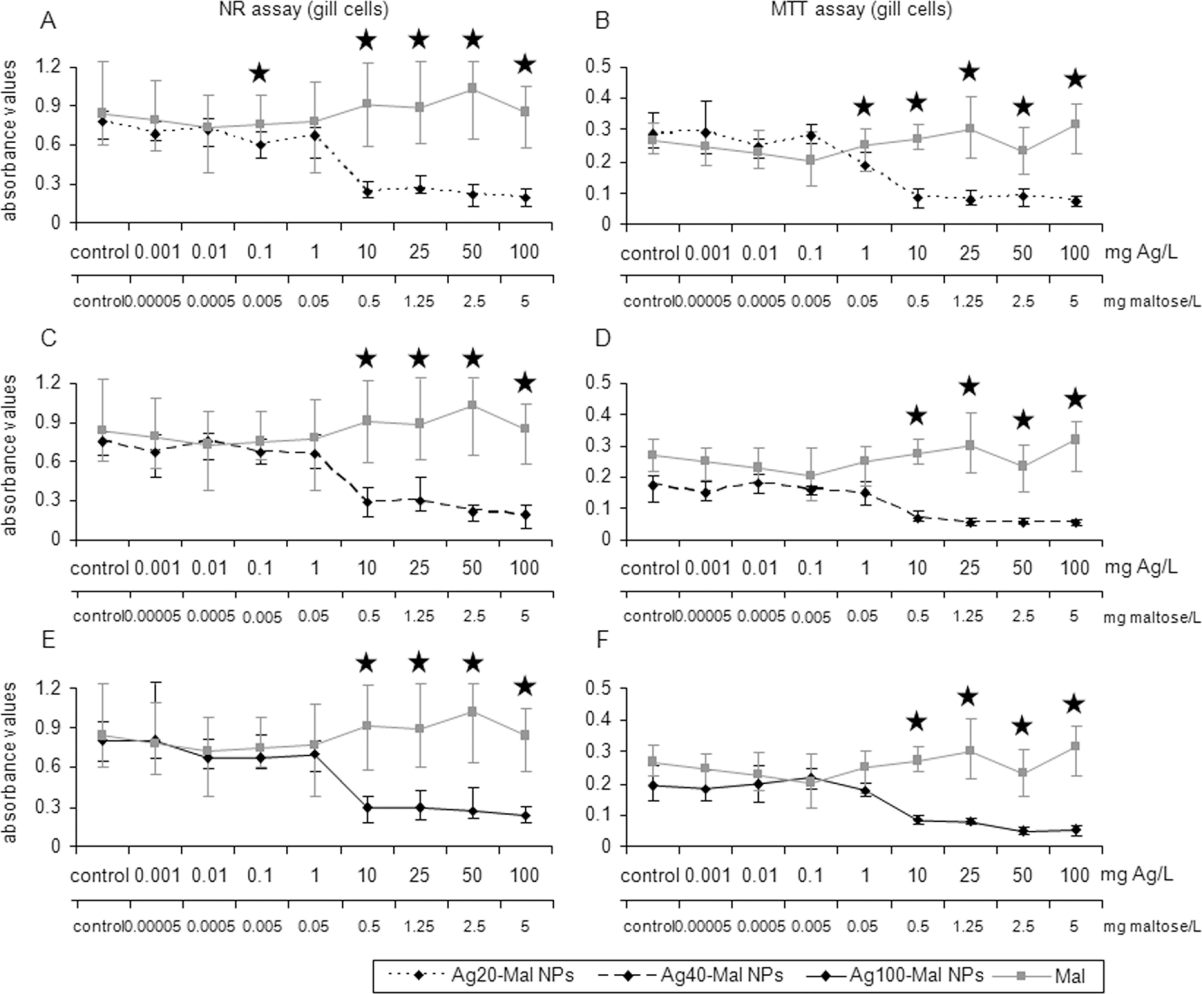
Effects of maltose-stabilized Ag NPs on gill cells viability. Cell viability based on NR and MTT assays in mussel gill cells exposed to Ag20-Mal (A and B), Ag40-Mal (C and D) and Ag100-Mal (E and F) NPs and to pure maltose (at the same concentrations present in corresponding maltose-stabilized Ag NPs suspensions) for 24 h. Data are given as absorbance values (means ± confidence intervals). Stars indicate significant differences (p<0.05) in treated cells with respect to controls according to the bootstrap analysis followed by Bonferroni’s correction. Black stars correspond to the maltose-stabilized Ag NPs and grey stars to the maltose results. *n* = 6 replicates per treatment.

The three sizes of maltose stabilized Ag NPs decreased hemocytes viability (52–70% decrease) at the same concentration of that in bulk Ag exposure (10 mg Ag/L) (*p*<0.05) (Fig 5). In gill cells, Ag20-Mal NPs slightly decreased cell viability at 0.1 mg Ag/L (23% decrease, NR assay) and then a more marked decrease started at 10 mg Ag/L for the two assays (69–71% decrease) (*p*<0.05) (Fig 6A and 6B). Ag40-Mal and Ag100-Mal NPs decreased gill cells viability (57–62% decrease) at concentrations starting from 10 mg Ag/L in the two cell viability assays (*p*<0.05) (Fig 6C–6F). When tested alone, maltose showed minor effects on hemocytes viability only at 0.01 and 0.1 mg/L (27% decrease, MTT assay) (*p*<0.05) (Fig 5B, 5D and 5F) whereas in gill cells maltose was not cytotoxic (*p*>0.05) (Fig 6). Although the cytotoxicity of maltose stabilized Ag NPs started approximately in the same range of Ag concentrations of that in bulk Ag exposures, LC50 values of hemocytes and gill cells were lower for maltose stabilized Ag NPs than for bulk Ag, showing that maltose stabilized Ag NPs were more toxic than the bulk form (Table 1). Comparing the LC50 values of the three sizes of maltose stabilized Ag NPs, smaller NPs (Ag20-Mal) were more toxic than larger ones (Ag40-Mal and Ag100-Mal NPs) (Table 1) indicating a size-dependent cytotoxic effect.

**Fig 7:**
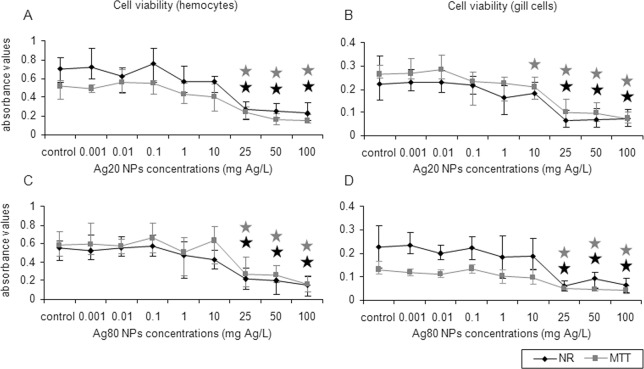
Effects of commercial Ag NPs on mussel cells viability. Cell viability (NR and MTT assays) in mussel hemocytes and gill cells exposed to Ag20 (A and B) and Ag80 (C and D) NPs for 24 h. Data are given as absorbance values (means ± confidence intervals). Stars indicate significant differences (p<0.05) in treated cells with respect to controls according to the bootstrap analysis followed by Bonferroni’s correction. Black stars correspond to the NR assay and grey stars to the MTT assay. *n* = 6 replicates per treatment.

Commercial Ag20 and Ag80 NPs were the least toxic NPs tested (Fig 7). Cytotoxicity of Ag20 NPs started at 25 mg Ag/L in hemocytes (52–62% decrease) and at 10 mg Ag/L (22% decrease, MTT assay) or 25 mg Ag/L (71% decrease, NR assay) in gill cells (*p*<0.05) (Fig 7A and 7B). Ag80 NPs decreased cell viability (52–72% decrease) starting at 25 mg Ag/L in both cell types and for the two cell viability assays (*p*<0.05) (Fig 7C and 7D). Based on the LC50 values, commercial Ag NPs showed a similar cytotoxicity than that of bulk Ag (Table 1). Comparing the LC50 values obtained in the different Ag NP exposures, Ag20-Mal NPs were the most cytotoxic NPs tested for the two cell types (Table 1) and thus, they were selected for in-depth mechanistic studies at sublethal doses (below LC25 values). As maltose provoked minor cytotoxic effects in mussel cells, maltose alone was not tested in the mechanistic assays.

### Mechanistic tests

**Fig 8:**
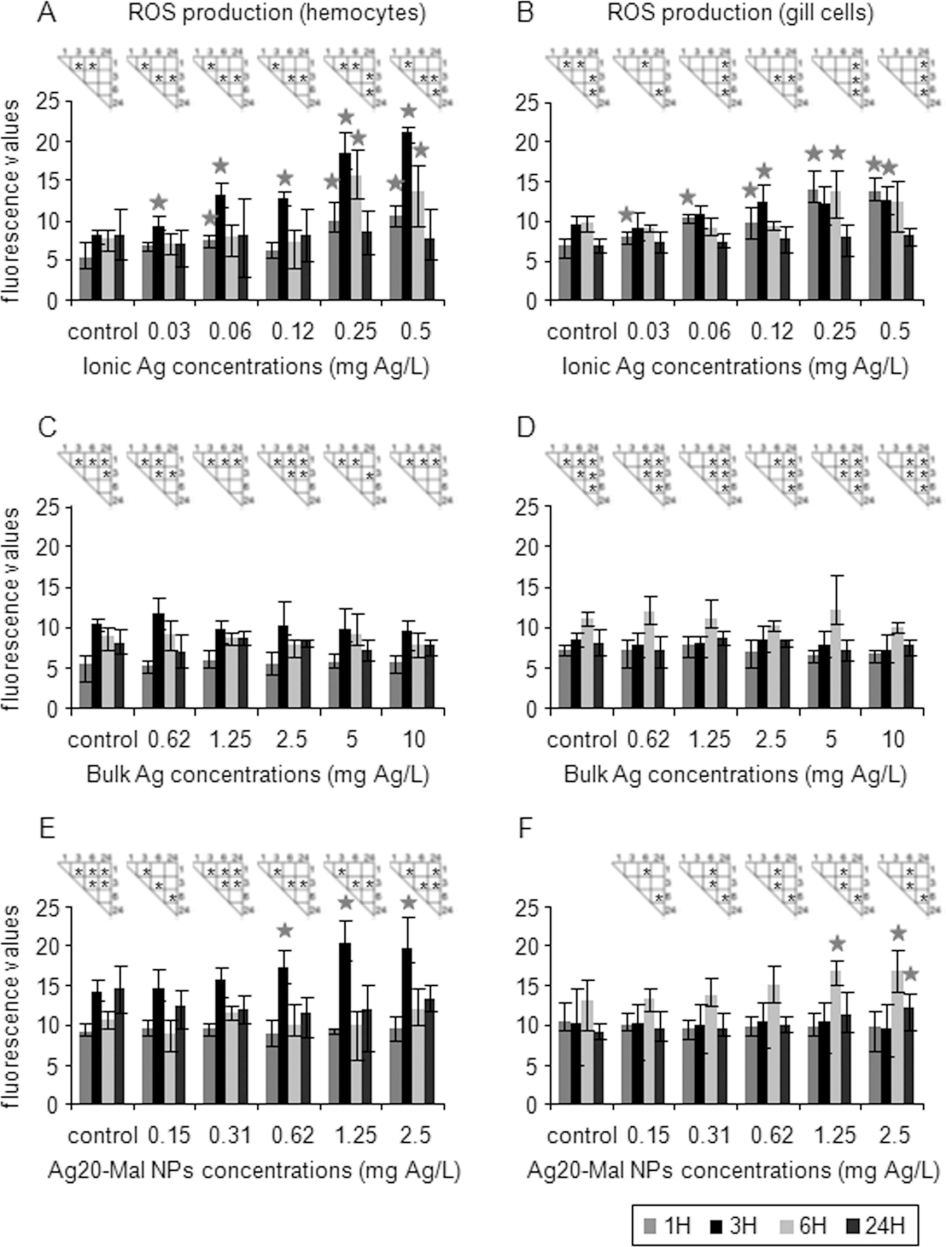
Effects of ionic Ag, bulk Ag and Ag NPs on ROS production in mussel cells. ROS production in mussel hemocytes and gill cells exposed to ionic Ag (A and B), bulk Ag (C and D) and Ag20-Mal NPs (E and F) for 1, 3, 6, and 24 h. Data are given as fluorescence values (means ± confidence intervals). Asterisks indicate significant differences (p<0.05) between times of exposure and stars indicate significant differences (p<0.05) in treated cells with respect to controls according to the bootstrap analysis followed by Bonferroni’s correction. *n* = 6 replicates per treatment.

At sublethal doses, the three forms of Ag altered a diverse range of cellular processes in hemocytes and gill cells. ROS production increased significantly in the two cell types exposed to ionic Ag and Ag20-Mal NPs whereas no effect on ROS was detected in exposures to bulk Ag (Fig 8). Production of ROS was monitored at 1, 3, 6, and 24 h and significant differences over time were observed in all exposures (Fig 8). Ionic Ag increased ROS production in both cell types starting at 0.03 mg Ag/L (16% increase) (*p*<0.05) (Fig 8A and 8B). ROS levels remained high until 6 h exposure in both cell types but decreased to control levels after 24 h (Fig 8A and 8B). The peak of ROS production was at 3 h in hemocytes and at 1–3 h in gill cells (Fig 8A and 8B). Ag20-Mal NPs increased ROS production in hemocytes (21% increase) exposed for 3 h to concentrations starting at 0.62 mg Ag/L (*p*<0.05) (Fig 8E). In gill cells, Ag20-Mal NPs increased ROS production (28% increase) at 6 h exposure to 1.25 mg Ag/L and at 6 and 24 h exposure to 2.5 mg Ag/L (27–31% increase) (*p*<0.05) (Fig 8E and 8F).

**Fig 9:**
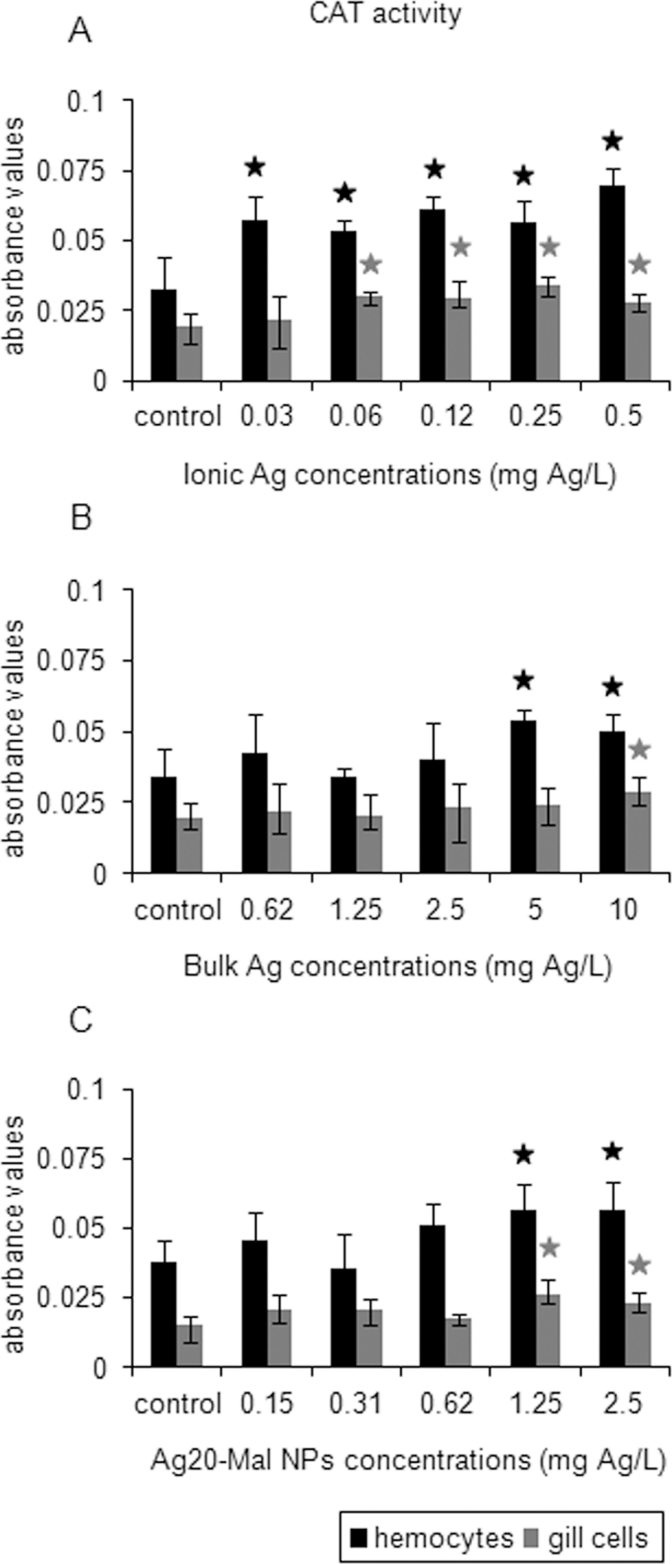
Effects of ionic Ag, bulk Ag and Ag NPs on CAT activity in mussel cells. CAT activity in mussel hemocytes and gill cells exposed to ionic Ag (A), bulk Ag (B) and Ag20-Mal NPs (C) for 24 h. Data are given as absorbance values (means ± confidence intervals). Stars indicate significant differences (p<0.05) in treated cells with respect to controls according to the bootstrap analysis followed by Bonferroni’s correction. *n* = 4 replicates per treatment.

The three forms of Ag increased CAT activity in hemocytes and gill cells (Fig 9). Ionic Ag increased CAT activity starting at 0.03 mg Ag/L exposure in hemocytes (76% increase) and starting at a higher concentration (0.06 mg Ag/L) in gill cells (55% increase) (*p*<0.05) (Fig 9A). In bulk Ag exposures, CAT activity increased at 5 and 10 mg Ag/L in hemocytes and at 10 mg Ag/L in gill cells (48–58% increase) (*p*<0.05) (Fig 9B). Ag20-Mal NPs increased CAT activity of both cell types at 1.25 and 2.5 mg Ag/L (48–75% increase) (*p*<0.05) (Fig 9C).

**Fig 10:**
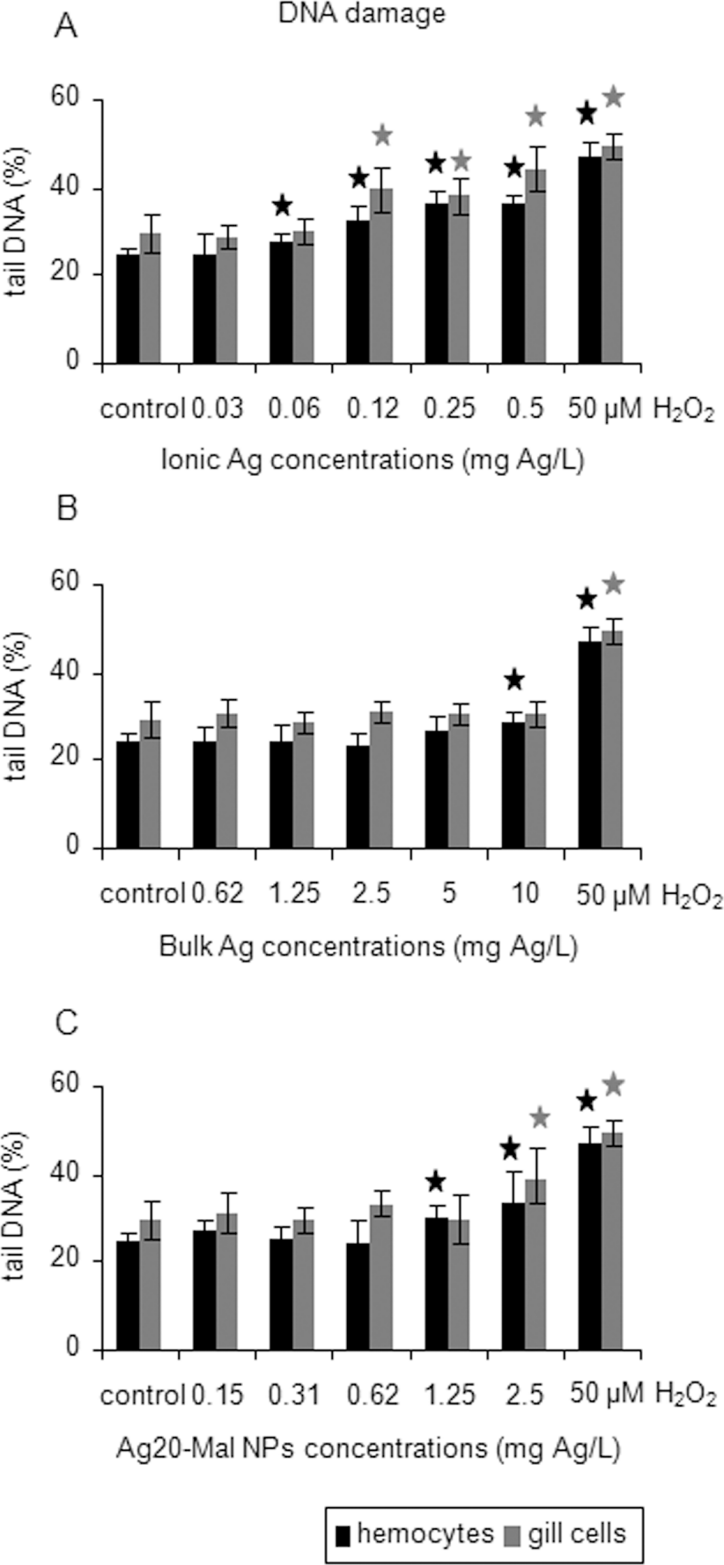
DNA damage in mussel cells exposed to ionic Ag, bulk Ag and Ag NPs. Results of the Comet assay in mussel hemocytes and gill cells exposed to ionic Ag (A), bulk Ag (B) and Ag20-Mal NPs (C) for 24 h. Data are given as arbitrary values for tail DNA (means ± confidence intervals). Cells exposed to 50 μM H_2_O_2_ were used as positive control. Stars indicate significant differences (p<0.05) in treated cells with respect to controls according to the bootstrap analysis followed by Bonferroni’s correction. *n* = 50 cells analyzed per treatment.

DNA damage was found in hemocytes exposed to the three forms of Ag and in gill cells exposed to ionic Ag and Ag20-Mal NPs (Fig 10). Hydrogen peroxide (50 μM), used as positive control in the Comet assay, showed the highest levels of DNA damage in both cell types (Fig 10). Ionic Ag produced DNA damage starting at 0.06 mg Ag/L exposure in hemocytes (12% increase) and starting at a higher concentration (0.12 mg Ag/L) in gill cells (35% increase) (*p*<0.05) (Fig 10A). Bulk Ag was genotoxic only in hemocytes exposed to the maximum concentration tested (10 mg Ag/L) (16% increase) (*p*<0.05) (Fig 10B). Ag20-Mal NPs produced DNA damage at 1.25 and 2.5 mg Ag/L in hemocytes (22–37% increase) and at 2.5 mg Ag/L in gill cells (32% increase) (*p*<0.05) (Fig 10C).

**Fig 11:**
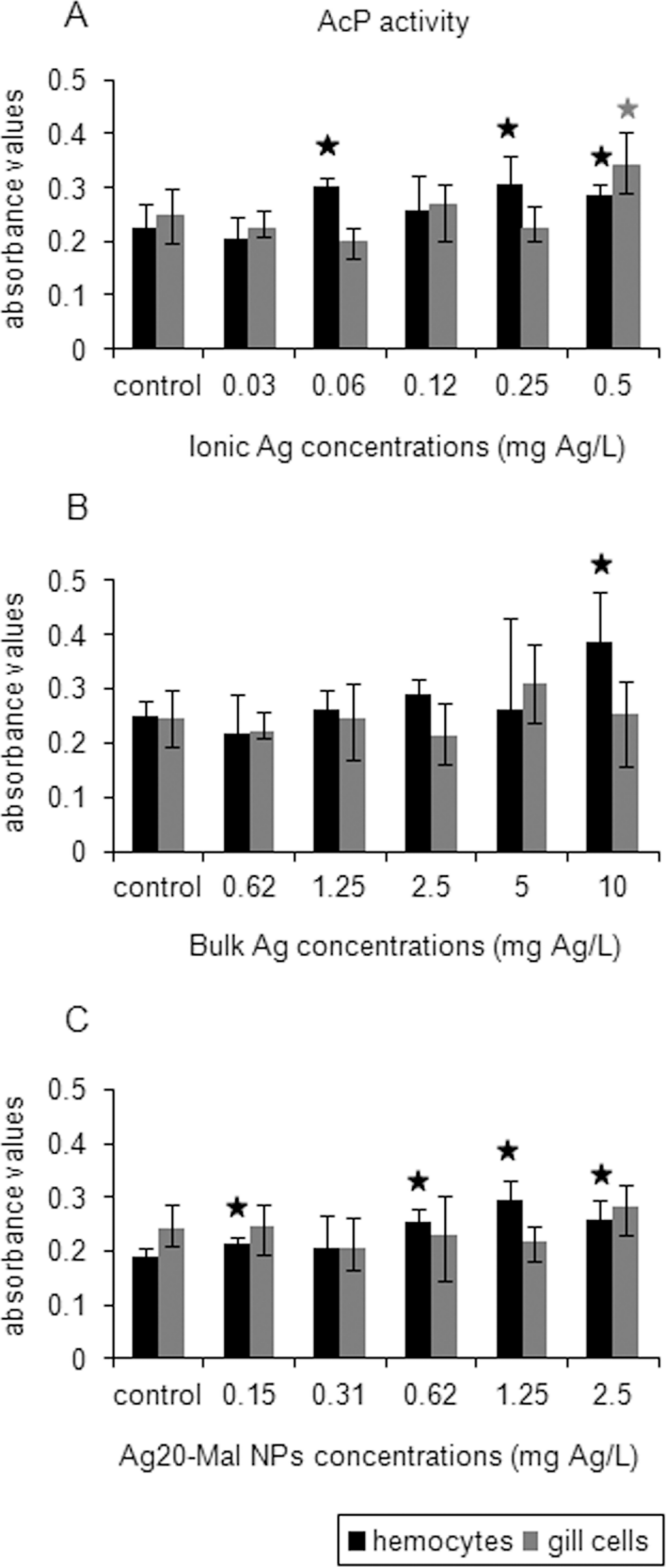
Effects of ionic Ag, bulk Ag and Ag NPs on AcP activity in mussel cells. Lysosomal AcP activity in mussel hemocytes and gill cells exposed to ionic Ag (A), bulk Ag (B) and Ag20-Mal NPs (C) for 24 h. Data are given as absorbance values (means ± confidence intervals). Stars indicate significant differences (p<0.05) in treated cells with respect to controls according to the bootstrap analysis followed by Bonferroni’s correction. *n* = 6 replicates per treatment.

Lysosomal AcP activity increased in hemocytes exposed to the three forms of Ag, whereas in gill cells, AcP activity increased only in ionic Ag exposure (Fig 11). In ionic Ag exposures, hemocytes AcP activity increased at 0.06, 0.25 and 0.5 mg Ag/L, (26–37% increase) while in gill cells AcP activity increased only when exposed to the maximum concentration tested (0.5 mg Ag/L) (37% increase) (*p*<0.05) (Fig 11A). Bulk Ag increased hemocytes AcP activity only at 10 mg Ag/L (53% increase) (*p*<0.05) (Fig 11B). Ag20-Mal NPs increased hemocytes AcP activity at 0.15 mg Ag/L and at higher doses (0.62–2.5 mg Ag/L) (13–58% increase) (*p*<0.05) (Fig 11C).

**Fig 12:**
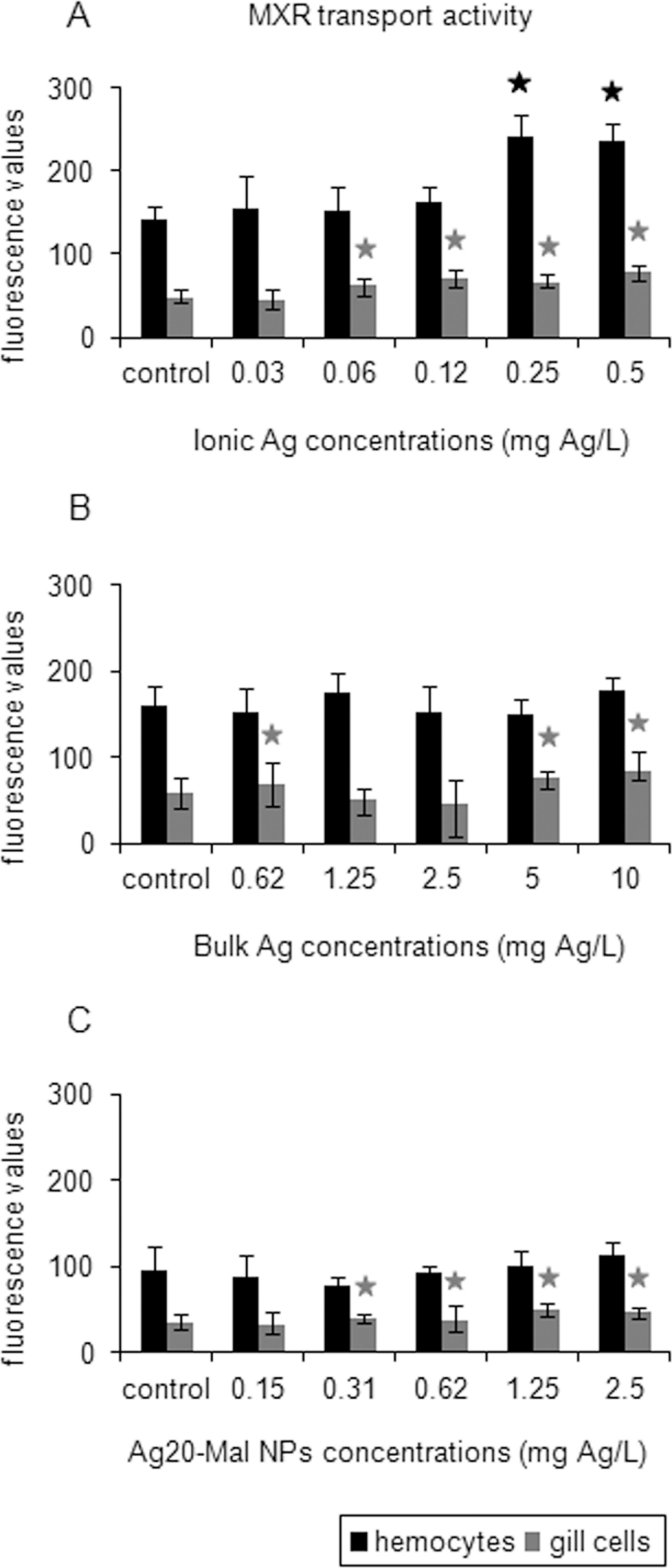
Effects of ionic Ag, bulk Ag and Ag NPs on MXR activity in mussel cells. MXR transport activity in mussel hemocytes and gill cells exposed to ionic Ag (A), bulk Ag (B) and Ag20-Mal NPs (C) for 24 h. Data are given as fluorescence values (means ± confidence intervals). Stars indicate significant differences (p<0.05) in treated cells with respect to controls according to the bootstrap analysis followed by Bonferroni’s correction. *n* = 6 replicates per treatment.

MXR transport activity increased in gill cells exposed to the three forms of Ag while in hemocytes MXR activity increased only in ionic Ag exposure (Fig 12). Ionic Ag increased hemocytes MXR activity at 0.25 and 0.5 mg Ag/L (60% increase) (*p*<0.05) (Fig 12A). In gill cells, MXR activity increased when exposed to concentrations starting at 0.06 mg Ag/L (30–59% increase) (*p*<0.05) (Fig 12A). Bulk Ag increased gill cells MXR activity at 0.62 mg Ag/L and then at 5 and 10 mg Ag/L (16–42% increase) (*p*<0.05) (Fig 12B). Ag20-Mal NPs increased gill cells MXR activity at concentrations starting at 0.31 mg Ag/L (14–35% increase) (*p*<0.05) (Fig 12C).

**Fig 13:**
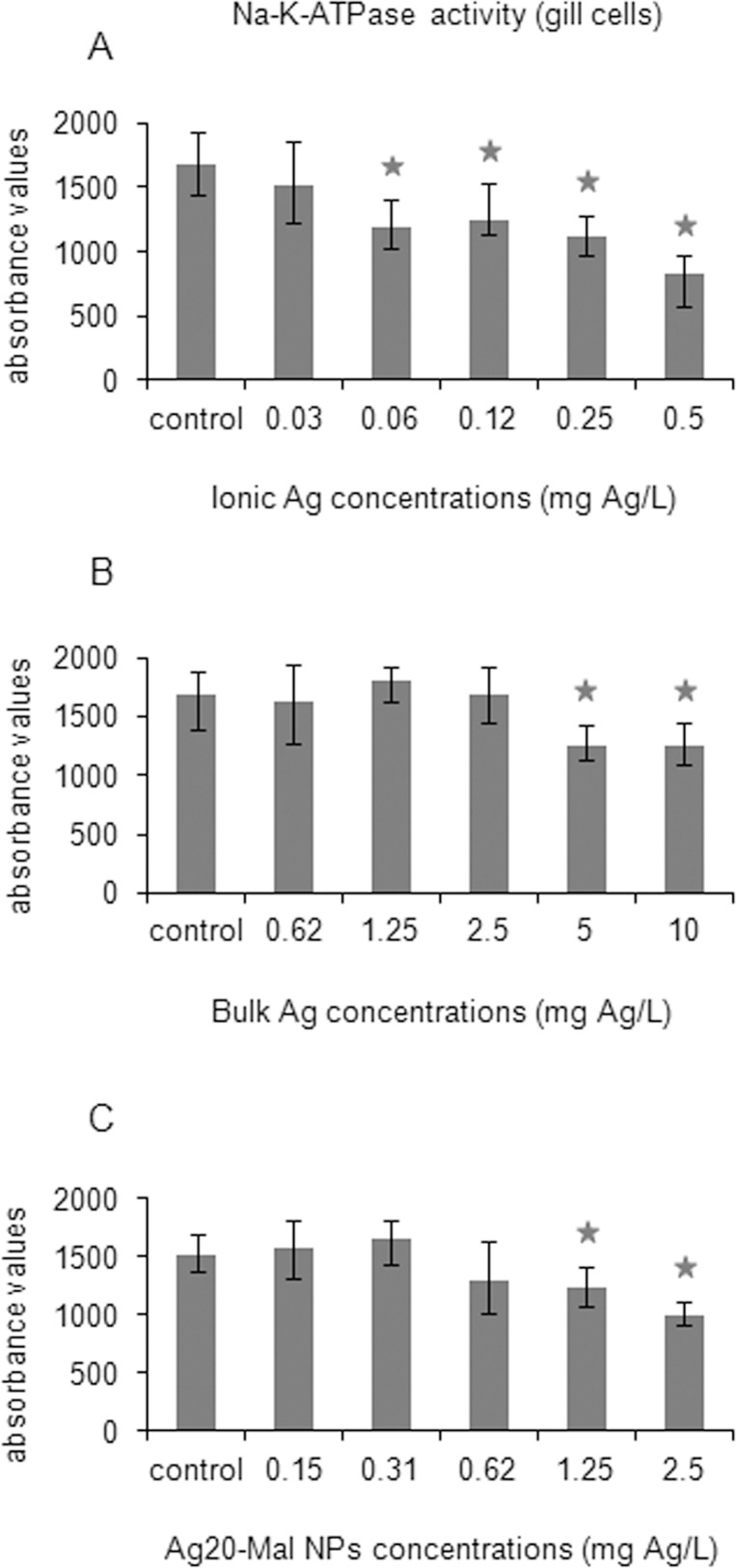
Effects of ionic Ag, bulk Ag and Ag NPs on Na-K-ATPase activity in mussel cells. Na-K-ATPase activity in mussel gill cells exposed to ionic Ag (A), bulk Ag (B) and Ag20-Mal NPs (C) for 24 h. Data are given as absorbance values (means ± confidence intervals). Stars indicate significant differences (p<0.05) in treated cells with respect to controls according to the bootstrap analysis followed by Bonferroni’s correction. *n* = 6 replicates per treatment.

All three forms of Ag decreased Na-K-ATPase activity in gill cells (Fig 13). Decreases in this enzyme activity were found starting at 0.06 mg Ag/L in ionic Ag exposure (30% decrease) (Fig 13A), at 5 mg Ag/L in bulk Ag exposure (26% decrease) (Fig 13B) and at 1.25 mg Ag/L in Ag20-Mal NPs exposure (Fig 13C) (19% decrease) (*p*<0.05).

**Fig 14:**
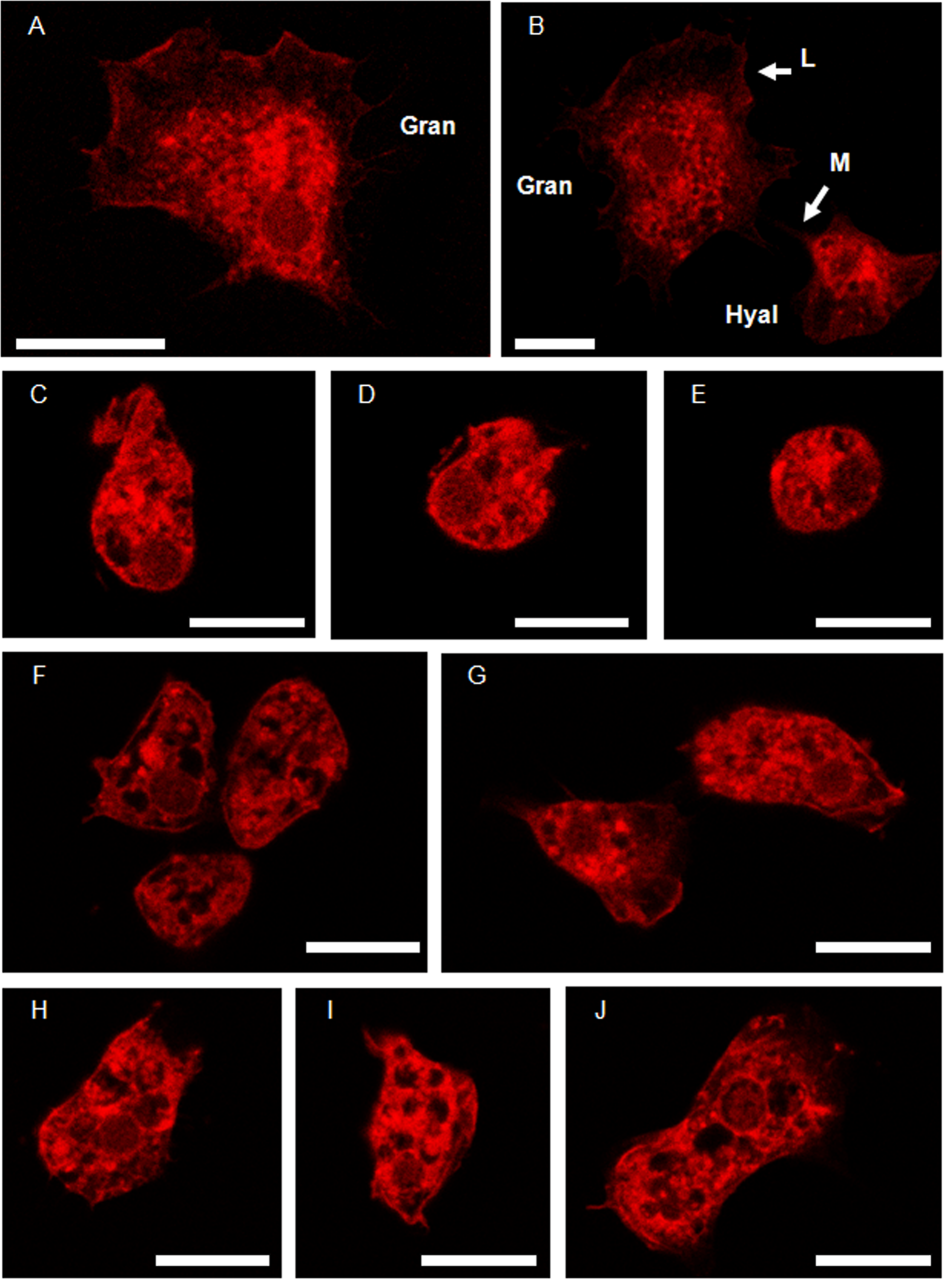
Effects of ionic Ag, bulk Ag and Ag NPs on the actin cytoskeleton of hemocytes. Actin cytoskeleton (labelled with TRITC-conjugated phalloidin) of untreated mussel hemocytes (A and B) and hemocytes treated with ionic Ag (C-E), bulk Ag (F and G) and Ag20-Mal NPs (H-J). Hyal, hyalinocyte; Gran, granulocyte; L, lamellipodia; M, microspikes. Scale bars = 20 μm.

Alterations in the actin cytoskeleton integrity were found in hemocytes treated with the different Ag forms compared to control cells (Fig 14). In untreated cells labelled with TRITC-conjugated phalloidin two types of hemocytes (hyalinocytes and granulocytes) were readily distinguished (Fig 14A and 14B). Hyalinocytes showed actin-containing microspikes (Fig 14B) while granulocytes showed extended lamellipodia (Fig 14A and 14B) forming the leading edge of migrating cells. Stronger effects were found in hemocytes exposed to ionic Ag (Fig 14C–14E) comparing to exposures to bulk Ag (Fig 14F and 14G) and to Ag20-Mal NPs (Fig 14H–14J). Hemocytes exposed to ionic Ag showed a rounded shape that could indicate cells detachment from the wells (Fig 14C–14E). Reduction in microspikes and lamellipodia were observed in hemocytes exposed to bulk Ag and to Ag20-Mal NPs (Fig 14F–14J).

**Fig 15:**
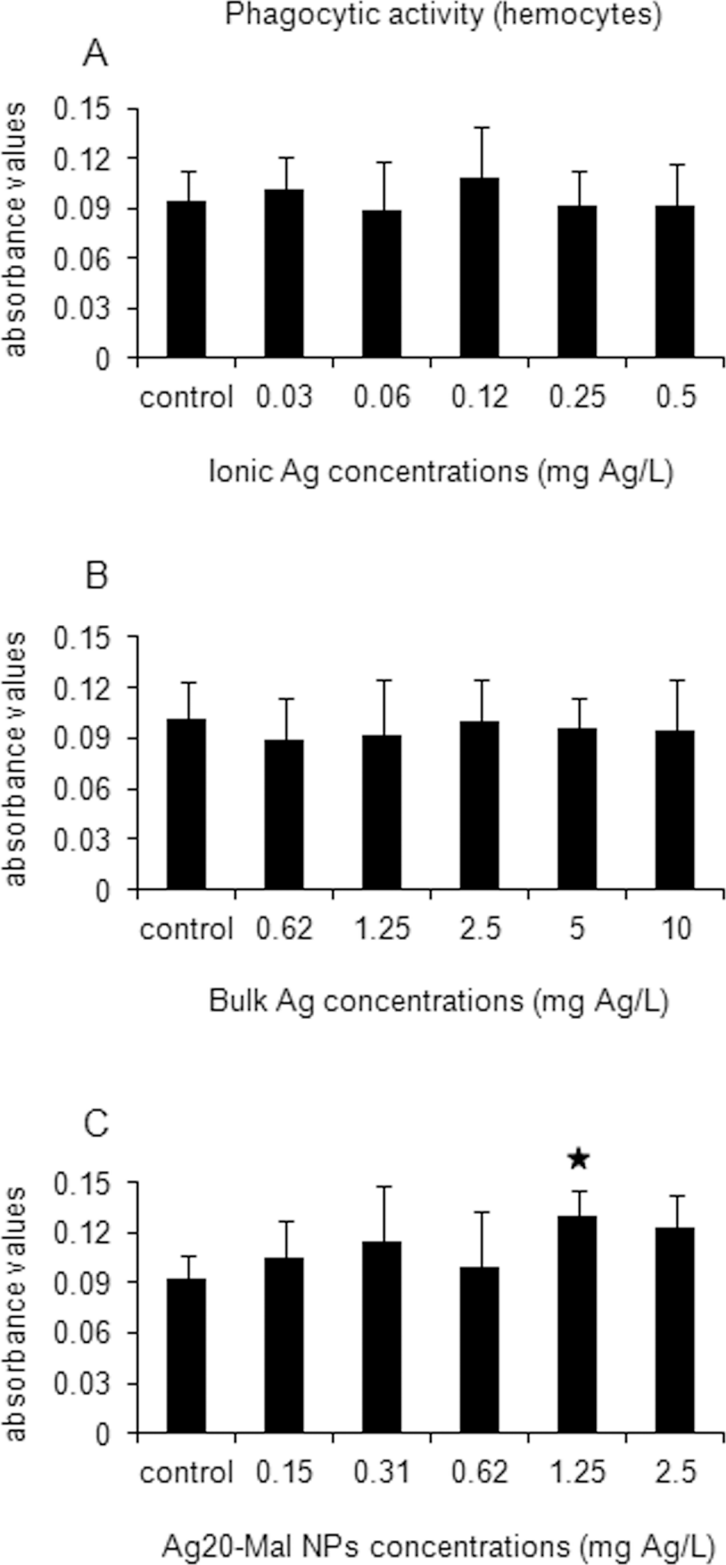
Effects of ionic Ag, bulk Ag and Ag NPs on phagocytic activity in hemocytes. Phagocytic activity in mussel hemocytes exposed to ionic Ag (A), bulk Ag (B) and Ag20-Mal NPs (C) for 24 h. Data are given as absorbance values (means ± confidence intervals). Stars indicate significant differences (p<0.05) in treated cells with respect to controls according to the bootstrap analysis followed by Bonferroni’s correction. *n* = 6 replicates per treatment.

Minor effects were found in the hemocytes phagocytic activity (Fig 15). Hemocytes phagocytic activity increased significantly only when exposed to 1.25 mg Ag/L of Ag20-Mal NPs (41% increase) (*p*<0.05) (Fig 15C).

## Discussion

The mechanisms underlying the toxicity of different NPs in marine organisms are still poorly understood. *In vitro* techniques have been demonstrated to be valuable tools to detect the toxicity of NPs and to identify cellular mechanisms altered by the exposure to NPs. However, few studies have been published trying to elucidate the mechanisms of action of different NPs in mussel cells *in vitro* [21, 22, 24, 27] and none of them have dealt with the effects of Ag NPs.

The characterization of the physico-chemical properties of Ag NPs is essential for understanding their behavior in different media and their potential toxicity. In the present work, maltose-stabilized Ag NPs, commercial Ag NPs and bulk Ag showed different physico-chemical characteristics that correlated with their relative toxicity. Maltose-stabilized Ag NPs were monodispersed particles, whereas the commercial Ag NPs showed a polydispersed distribution with some aggregates of NPs. Bulk Ag showed particles over 2 μm in size. Maltose-stabilized Ag NPs, commercial Ag NPs and bulk Ag showed different zeta potential values but in all cases samples showed highly charged surfaces (high zeta potential values ranging from -30 to -68 mV), corresponding to colloidally stable samples. Maltose-stabilized Ag NPs showed a rapid and continuous dissolution in saline media. For the three sizes of maltose-stabilized Ag NPs tested, over 20% of the total Ag was converted into Ag ions after 168 h. Moreover, there appeared to be some influence of particle size, with the smaller Ag20-Mal NPs clearly having the highest rate of dissolution presumably due to the higher relative surface area. These findings are in agreement with previous studies showing that dissolution of Ag NPs varies with NPs size and with different surface coatings [46–48]. For the *in vitro* tests, cells were exposed for 24 h and thus, approximately 10% of maltose-stabilized Ag NPs would be dissolved (11.7, 8.7 and 7.2% for Ag20-Mal, Ag40-Mal and Ag100-Mal in seawater, respectively). These results are in line with those reported by Burchardt et al. [49], who suggested that toxicity of Ag NPs in the marine diatom *Thalassiosira pseudonana* resulted from a shared effect of the particles toxicity and the release of Ag ions.

Marked differences were found comparing the toxicity of ionic Ag, bulk Ag and Ag NPs to mussel hemocytes and gill cells. Generally, similar results were obtained for the two cell types but gill cells were slightly more sensitive than hemocytes (see Table 2). Ionic Ag was the most toxic form tested in the cell viability assays and also in the mechanistic tests. It is well known that ionic Ag is one of the most toxic metals for aquatic organisms and that invertebrates are sensitive to very low concentrations of this metal [4]. Other studies have also shown that ionic Ag is more toxic than Ag NPs *in vivo* [11, 50, 51] and *in vitro* [15, 51–53]. Bulk Ag showed a relatively lower toxicity to mussel cells as well as in other studies with human cells [54] and fish cell lines [50]. Comparing the toxicity of maltose-stabilized Ag NPs and commercial Ag NPs, the first were more toxic whereas cytotoxicity of the commercial Ag NPs was comparable to that of the bulk form. Maltose did not appear to contribute significantly to the maltose-stabilized Ag NPs toxicity. Differences in the cytotoxicity of the maltose-stabilized Ag NPs compared to the commercial Ag NPs may be related to their intrinsic properties (state of dispersion of the NPs and the presence of aggregates), as mentioned earlier.

**Table 2:** Summary table of the and LC50 values (in mg Ag/L) obtained in hemocytes and gill cells exposed to ionic Ag, bulk Ag, Ag NPs (Ag20-Mal, Ag40-Mal, Ag100-Mal, Ag20, Ag80) and pure maltose based on NR and MTT assays.

	Hemocytes		Gill cells	
	NR	MTT	NR	MTT
**Ionic Ag**	**1.065**	**1.144**	**0.883**	**0.959**
**Bulk Ag**	**18.703**	**20.873**	**17.865**	**19.885**
**Ag20-Mal**	**4.74**	**5.803**	**4.395**	**4.835**
**Ag40-Mal**	**8.285**	**8.43**	**7.444**	**7.657**
**Ag100-Mal**	**9.03**	**9.503**	**7,63**	**8.691**
Maltose	>171.1 (> 100)	>171.1 (> 100)	>171.1 (> 100)	>171.1 (> 100)
**Ag20**	**22.47**	**22.749**	**18.224**	**20.45**
**Ag80**	**19.133**	**21.767**	**18.777**	**19.446**

The LC50 values of the maltose are given in mg maltose/L and in equivalent metal concentrations present in maltose-stabilized Ag NPs suspensions (in braquets).

Maltose-stabilized Ag NPs showed size-dependent cytotoxicity. Small NPs (Ag20-Mal) were significantly more toxic than the larger ones (Ag40-Mal and Ag100-Mal). Other studies have also reported size-dependent toxicity of Ag NPs [55–58]. The larger surface area could be contributing for their higher reactivity, related to their faster dissolution in the media, as mentioned before.

Compared with other *in vitro* studies, the range of concentrations that were cytotoxic for mussel cells (generally 10 to 100 mg Ag/L for hemocytes and 1 to 100 mg Ag/L for gill cells) were similar to those reported by other authors. George et al. [59] exposed the rainbow trout gill fish cell line RT-W1 to different Ag NPs and found that cytotoxicity started at 25 mg Ag/L. In the study of Taju et al. [51], decreases in cell viability (NR and MTT assays) were observed in fish gill cell lines exposed to AgNO_3_ and Ag NPs (2–64 mg/L) in a dose-dependent manner. Ag NPs were cytotoxic to rainbow trout hepatocytes starting at 19 mg Ag/L [60] and to gill cells starting at 5 mg Ag/L [15].

Several studies have indicated that cytotoxicity of Ag NPs is closely related to the increase in the production of ROS and triggering of the cellular antioxidant mechanisms [59, 61–67]. In agreement, in mussel hemocytes and gill cells, ROS production was significantly increased after exposures to ionic Ag and Ag20-Mal NPs. Interestingly, the peak of ROS production occurred earlier in hemocytes (3 h) than in gill cells (6 h), which could be related to the role of hemocytes in immune defense. The activity of the antioxidant enzyme CAT increased in both cell types exposed to the three forms of Ag. In ionic Ag and Ag20-Mal NP exposures, increases in ROS production and CAT activity occurred at similar concentrations. Ag ions play a role in catalyzing the production of ROS in the presence of oxygen species [68]. Interestingly, Ag NPs themselves can produce ROS and oxidative stress *in vitro*, as well as release of Ag ions [68]. The increase in ROS levels in mussel cells would consequently lead to enhanced CAT activity. McCarthy et al. [17] also found increases in CAT activity of gills from oysters exposed to Ag NPs. Similarly, Buffet et al. [9] reported significant increases in glutathione S-transferase, CAT and caspase 3-like activities in *S*. *plana* and *H*. *diversicolor* exposed to ionic Ag and Ag NPs. Increases in ROS production and CAT activity were also observed in mussel hemocytes and gill cells after *in vitro* exposure to CdS quantum dots [27].

Our results demonstrate that both ionic Ag and Ag20-Mal NPs are genotoxic to mussel hemocytes and gill cells. At the highest concentration tested (10 mg Ag/L), bulk Ag also produced DNA damage in hemocytes. These findings are in accordance with previous studies on the genotoxicity of ionic Ag and Ag NPs [9, 11, 69–75]. In invertebrates, Gomes et al. [11] reported that ionic Ag and Ag NPs induced DNA damage in mussel hemocytes, a time-response effect being evidenced. Authors also showed that ionic Ag provoked a higher genotoxicity than Ag NPs, which is the case in the present study *in vitro*. Buffet et al. [9] showed that ionic Ag and Ag NPs produce a significant increase of DNA damage in *H*. *diversicolor* compared to controls, but no differences were found between the two Ag forms. Conversely, in the bivalve species *S*. *plana*, genotoxicity was significantly higher in animals exposed to Ag NPs compared to those exposed to ionic Ag indicating that in *S*. *plana*, toxicity of Ag NPs is not only related with the release of metal ions. Differences between *M*. *galloprovincialis* and *S*. *plana* could be related to their different feeding behavior that could lead to differential uptake and handling of Ag NPs. Nevertheless, the fact that bulk Ag produced DNA damage and increased CAT activity similar to Ag NPs, suggests that these cellular responses are not only related with dissolved Ag ions.

Due to the strong affinity of Ag with sulfhydryl groups of essential enzymes and with phosphorus-containing bases, ionic Ag can interact with DNA directly by the formation of ROS, causing damage by covalent binding to DNA or by inhibiting DNA synthesis, thus preventing cell division and DNA replication [71]. Ag NPs can cause DNA damage through direct interaction with DNA since Ag NPs have been found accumulated in cell nuclei of both zebrafish embryos and human cells [69, 70] and/or by leading to mitochondrial dysfunction, increase in ROS production, release of pro-apoptotic proteins from the mitochondria which in turn, set off DNA damage and chromosome aberrations [69].

Concerning effects on the lysosomal AcP activity, significant differences were found comparing the two cell types tested. The three forms of Ag increased AcP activity in hemocytes whereas in gill cells only ionic Ag increased this enzyme activity. It is well known that mussel hemocytes have an important role in the detoxification of metals through their sequestration and accumulation in their endolysosomal system [76, 77]. Metal oxide NPs (TiO_2_, SiO_2_, ZnO and CeO_2_) decreased lysosomal membrane stability in mussel hemocytes [24]. Authors attributed this toxic effect in part to the extracellular dissolution of NPs releasing toxic ions but also to particles uptake, digestion within the acidic endosomal/lysosomal compartments and liberation of free toxic ions in lysosomes. CdS quantum dots accumulated inside of endocytic-lysosomal vesicles of mussel hemocytes and this was associated with an increase in AcP activity [27]. In the present work, the induction of AcP activity in mussel cells may be related with the internalization of free toxic Ag ions or by the degradation of NPs in the endocytic-lysosomal system or could be even reflecting an enhancement in lysosomal formation for detoxification purposes [78].

Whereas mussel hemocytes were more sensitive than gill cells regarding lysosomal AcP activity, gill cells were more sensitive regarding the MXR transport activity. In gill cells, the three forms of Ag induced the MXR transport activity while in hemocytes only ionic Ag exposure caused this induction. Induction of MXR activity has been described in hemocytes exposed to different xenobiotics [79, 80], in gill cells exposed to metals such as Cd, Cu and Hg [81, 82] and in both hemocytes and gill cells exposed to CdS quantum dots [27]. Results suggested that the MXR transport activity plays a role in the detoxification of Ag, potentially reducing the intracellular accumulation and toxicity of Ag. Another explanation could be that the normal physiological function of transmembrane proteins (e.g. ionic channels, porins or receptors) could be disrupted by Ag NPs on the surface membrane [83]. This extracellular effect could also explain the induction of MXR in gill cells exposed to bulk Ag.

It is well known that one of the main toxic effects of silver on aquatic organisms is the inhibition of Na-K-ATPase followed by disturbance of ion balance [84]. Silver ions are taken up by gill cells via proton-coupled Na^+^ channels [85] and may then block the ion transporter Na-K-ATPase. The binding of Ag ions to sulfhydryl groups of Na-K-ATPase was reported as the most likely mechanism that cause Na-K-ATPase inhibition [86]. Griffitt et al. [87] found downregulation of Na-K-ATPase genes in gills of zebrafish exposed to Ag NPs. In the present work, the three forms of Ag inhibited the activity of Na-K-ATPase in gill cells. Inhibition was stronger in gill cells exposed to ionic Ag, followed by exposure to Ag20-Mal NPs and to a lower extent by exposure to the bulk Ag. These differences in the degree of inhibition of Na-K-ATPase activity in mussel gill cells could be related at least in part with differences in Ag ion release from each of the three forms of Ag but a direct interaction of Ag NPs or bulk Ag with this membrane ion transporter can not be ruled out, as indicated before for MXR.

Finally, exposures to the three forms of Ag affected the integrity of the hemocytes actin cytoskeleton with stronger effects caused by ionic Ag followed by Ag20-Mal NPs and bulk Ag. The disruption of actin cytoskeleton has already been reported in hemocytes treated with other metals such as Cd [35, 41] and Cu [35, 88]. In exposures to ionic Ag and Ag NPs, Gomes et al. [12] reported that both Ag forms altered the expression of proteins related with cytoskeleton and cell structure in hemocytes and suggested that this effect is a cumulative effect of Ag ions released from the particles and other properties such as particle size and surface reactivity.

In hemocytes, the actin cytoskeleton is responsible for the formation of cell extensions such as lamellipodia and thin radial microspikes [41] and thus, plays a key role in the phagocytic process. A stimulatory effect on phagocytic activity was found only in hemocytes exposed to Ag20-Mal NPs at 1.25 mg Ag/L. Exposures to NPs such as CdS quantum dots [27] and TiO_2_ [24] are known to increase phagocytic activity in hemocytes. Thus, of all the different cellular processes studied in the present work, the immunostimulatory effect found in hemocytes was the only potentially nano-specific effect detected and could be related with interactions of NPs with hemocytes cellular membrane.

Taken together, results showed differences in the toxicity of ionic Ag, bulk Ag and Ag20-Mal NPs in the two cell types, partly as a result of their different ability to release Ag ions. Nevertheless, in many cases the three forms of Ag affected the same cellular processes at different doses and with different magnitude of responses. In Ag20-Mal NP exposures, ROS-mediated oxidative stress, activation of antioxidant mechanisms and genotoxicity were the main responses in both cell types. Mussel gill cells are the cell type of choice for addressing the effects of NPs on transport activity across the plasma membrane, such as MXR and Na-K-ATPase, whereas hemocytes are better suited to study alterations on the endolysosomal system.

## Conclusions

In conclusion, the three forms of Ag were cytotoxic to mussel hemocytes and gill cells. Differences in cytotoxicity were found comparing the two Ag NPs (maltose stabilized more toxic than commercial ones). Maltose stabilized Ag NPs showed size-dependent cytotoxicity being smaller NPs more toxic than the larger ones. Ionic Ag was the most cytotoxic Ag form tested whereas bulk Ag showed similar cytotoxicity than that of commercial Ag NPs. Gill cells were slightly more sensitive than hemocytes in the cell viability assays and maltose did not contribute to the NPs toxicity. Main mechanisms of toxicity of Ag20-Mal NPs involved the increase of ROS production, CAT activity and DNA damage in both cell types, activation of lysosomal AcP activity, disruption of actin cytoskeleton and estimulation of phagocytosis in hemocytes and the increase of MXR transport activity and inhibition of Na-K-ATPase in gill cells. Similar effects were observed after exposure to ionic and bulk Ag in the two cell types, although generally ionic Ag was the most toxic form. Results suggest that observed responses in mussel hemocytes and gill cells were due in part to dissolved Ag, except for the stimulatory effect on hemocyte phagocytic activity that was found only in hemocytes exposed to 1.25 mg Ag/L maltose stabilized 20 nm Ag NPs. Further studies are necessary to quantitatively identify the contribution of released Ag ions and Ag NPs to observed toxic effects. This question might be addressed by separating the dissolved fraction from the NPs and then exposing cells to the two fractions separately, as in Li et al. [89] for ZnO NPs. Another possibility could be to assess the toxicity of NPs with a limited release of ions.
